# Supplementary figures and images for: Plasmodium falciparum importation does not sustain malaria transmission in a semi-arid region of Kenya
Source: PLOS Glob Public Health. 2022 Aug 10;2(8):e0000807. doi: 10.1371/journal.pgph.0000807 (PMC10021402; doi:10.1371/journal.pgph.0000807)

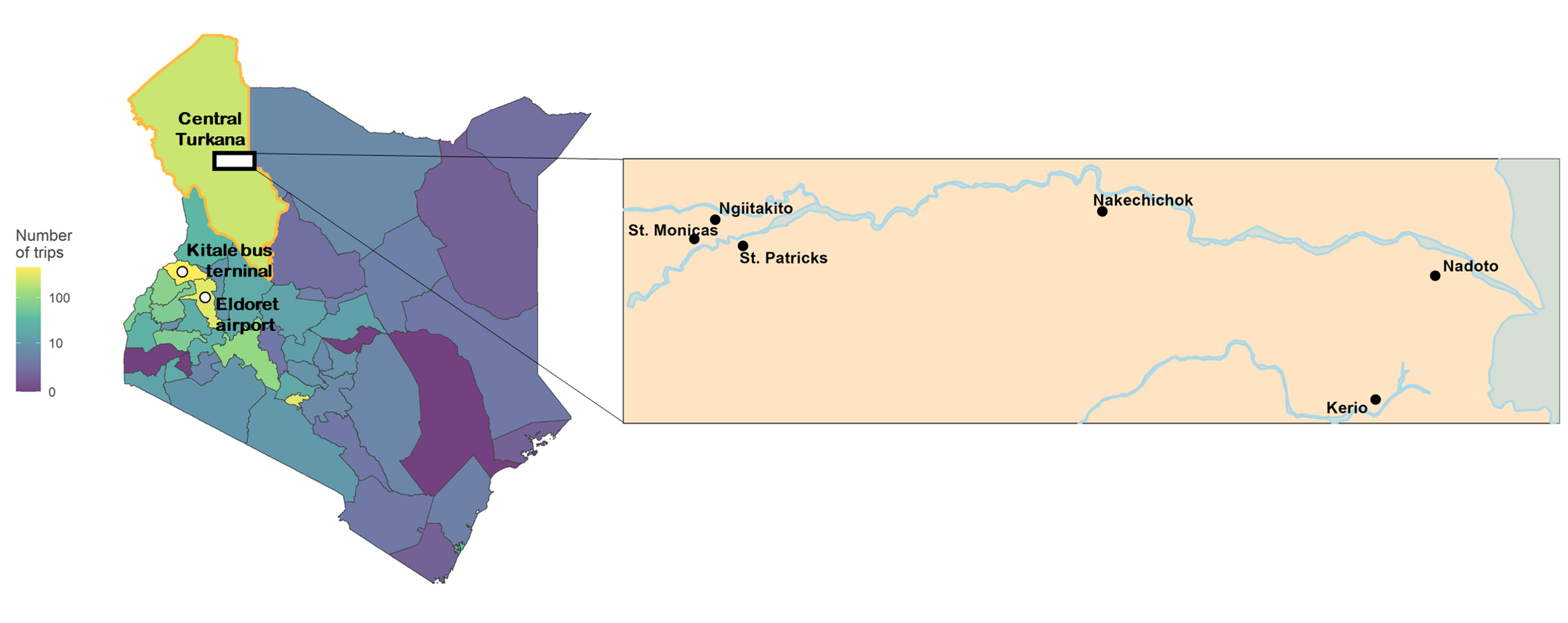

Supplement: S1 Fig — Turkana County is outlined in orange. County fill color denotes the number of trips to or from the designated county. The Kitale bus terminal and Eldoret airport are marked with white points. The study area in Central Turkana is highlighted with an inset map of health facilities to which index cases reported. Shapefile for the map of Kenya and county borders obtained from the Humanitarian Data Exchange (HDX), an open platform for sharing data across crises and organizations: https://data.humdata.org/dataset/cod-ab-ken. Inset map was created in QGIS (v 3.6.2-Noosa. Free and Open Source. QGIS Association. http://www.qgis.org. (TIF) [file pgph.0000807.s001.tif]

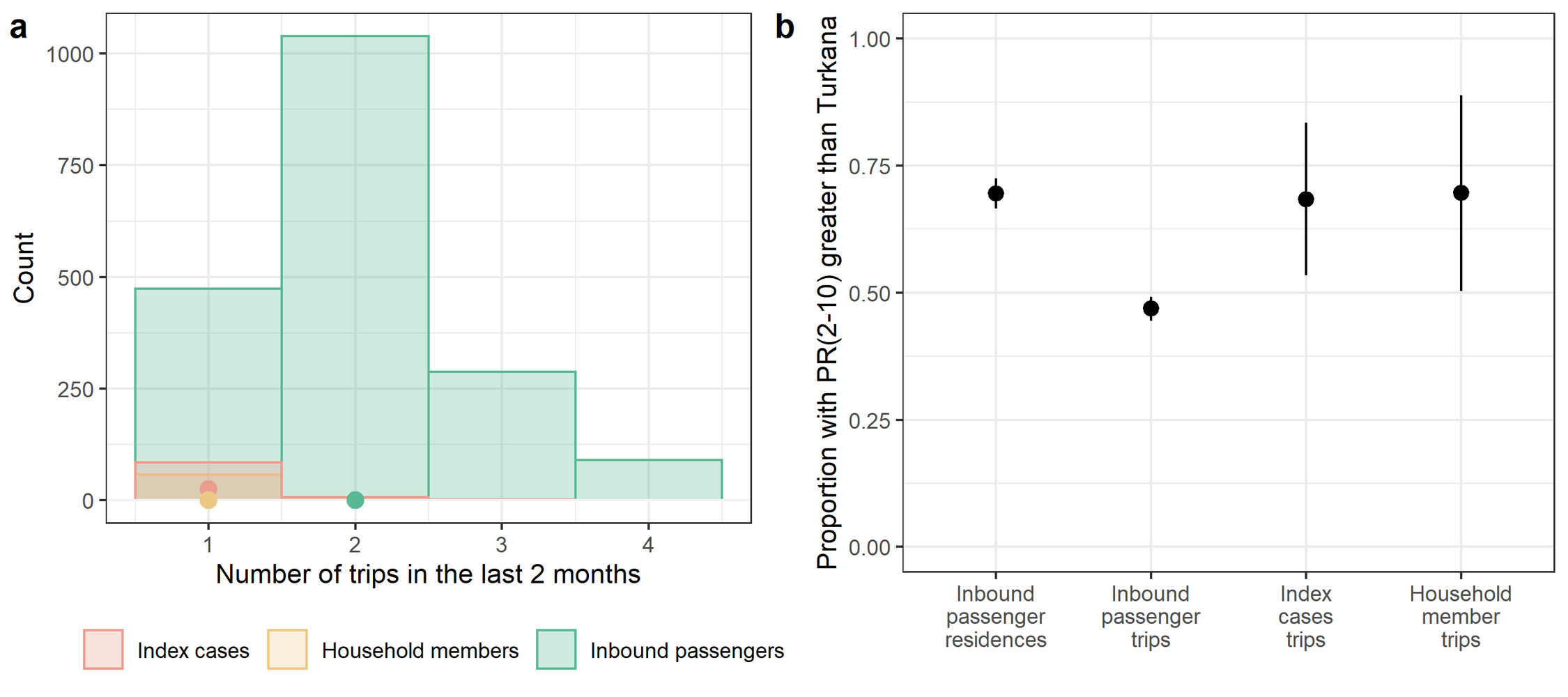

Supplement: S2 Fig — (a) Number of trips reported by travelers. Note that survey limited the number of trips from the two months prior to enrollment to 3, and the number of trips for inbound passengers includes the trip that led to enrollment. Points represent median values. (b) Proportion of trips and residences (of inbound passengers) with P. falciparum parasite rate in children ages 2–10 (PR(2–10)) in 2018–2019 greater than that of Turkana. PR(2–10) obtained from the Malaria Atlas Project [26]. Trips to/within Turkana excluded. (TIF) [file pgph.0000807.s002.tif]

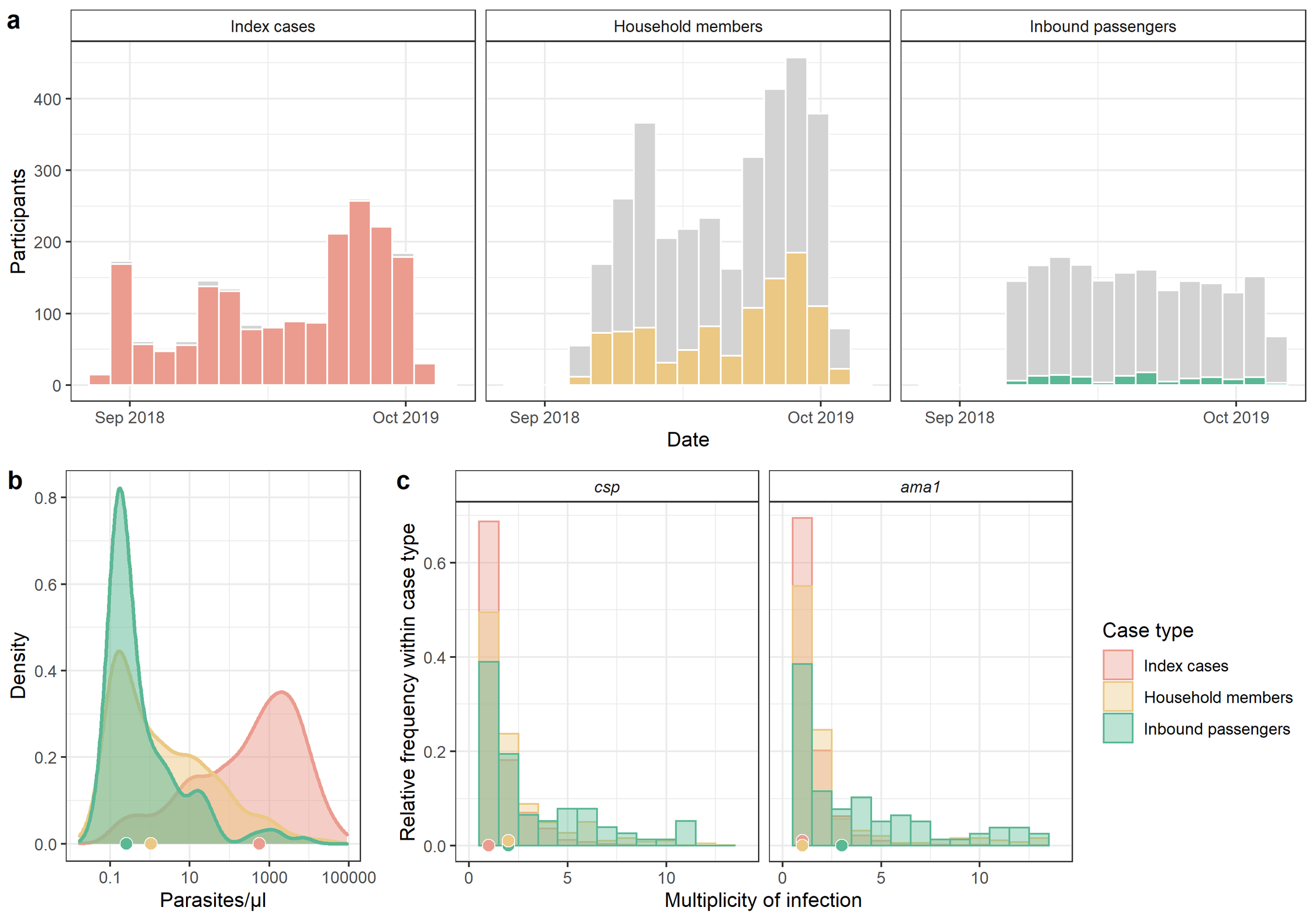

Supplement: S3 Fig — (a) PCR-positive (colored) and negative (grey) participants by study month and case type. (b) Parasite density distribution by case type. Points on x-axis represent median parasitemias for each case type. (c) Multiplicity of infection determined by csp (left) and ama1 (right) haplotype counts in each individual. Points on x-axis represent median MOIs for each case type. (TIF) [file pgph.0000807.s003.tif]

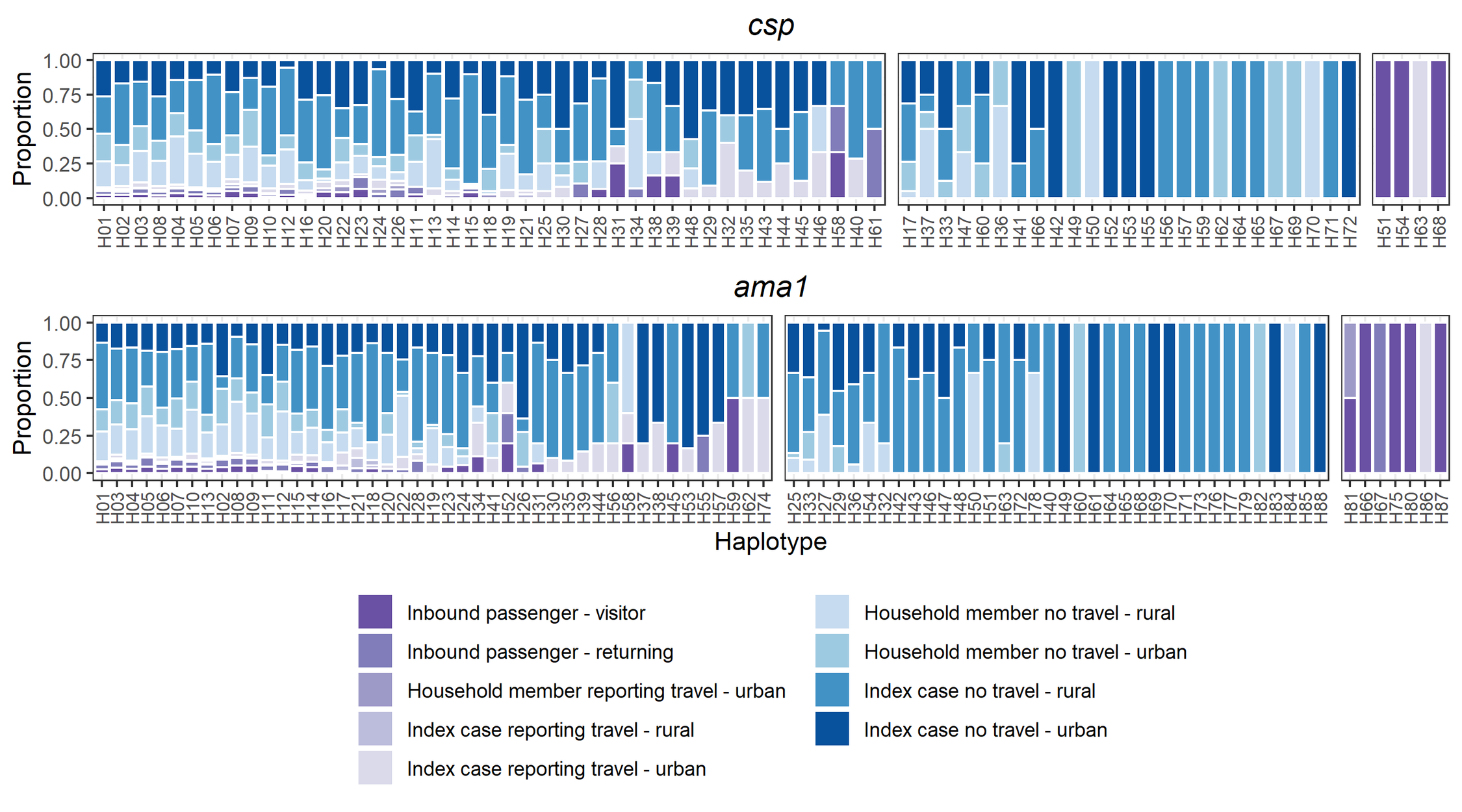

Supplement: S4 Fig — (TIF) [file pgph.0000807.s004.tif]

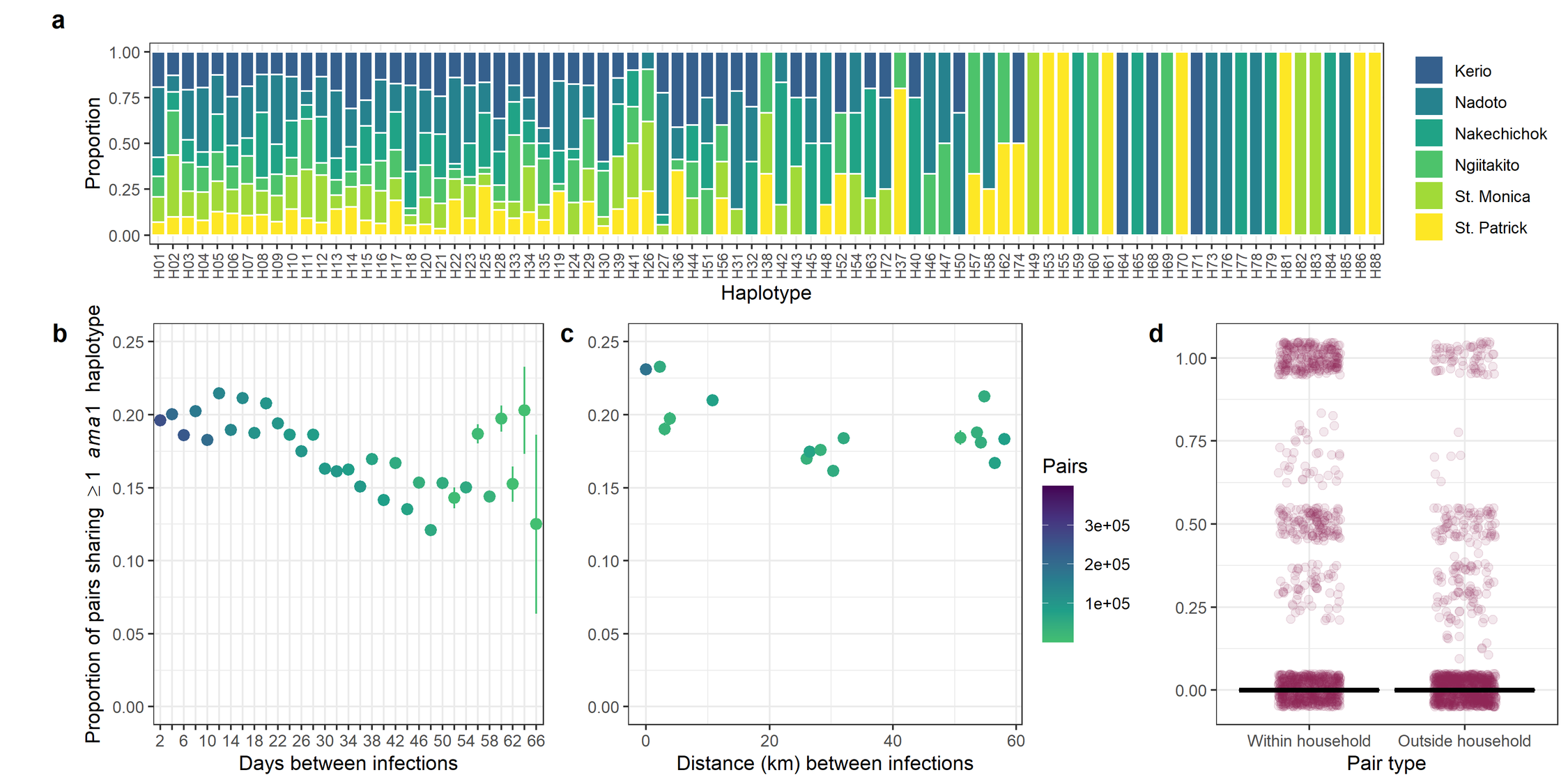

Supplement: S5 Fig — (a) A majority of ama1 haplotypes were detected at multiple sites across Central Turkana. Columns are individual ama1 haplotypes, and colors indicate the proportion of samples harboring the haplotypes detected at each of 6 enrollment sites. (b) The proportion of infection pairs sharing at least one ama1 haplotype decreases with increasing time between infections. Each dot represents the proportion of all pairs separated by the indicated interval (with n = the color) that shared at least 1 ama1 haplotype. (c) A marginal decrease in the proportion of individuals sharing at least one ama1 haplotype with increasing distance (facility level) between infections was observed. Each dot represents the proportion of all pairs separated by the indicated geographic distance (n = the color) that shared at least 1 ama1 haplotype. (d) The median proportion of individuals sharing at least one haplotype was greater within the household than outside the household. Within household, each dot represents the proportion of household members with whom an individual shared at least one haplotype. Outside household, each dot represents the median proportion of subsampled (n = number of household members, reps = 1000) infection pairs outside the household within ±60 days that share at least one haplotype. (TIF) [file pgph.0000807.s005.tif]

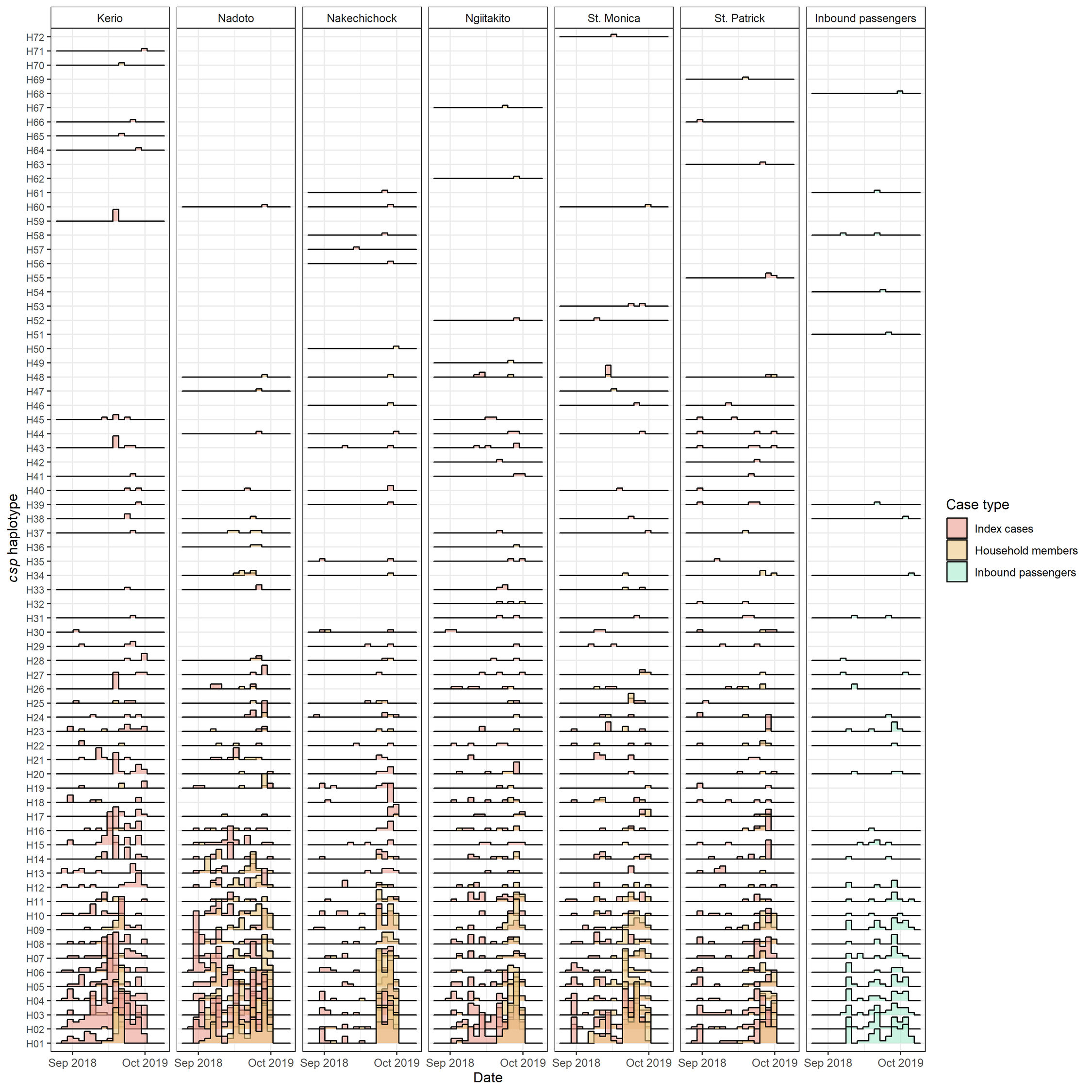

Supplement: S6 Fig — (TIF) [file pgph.0000807.s006.tif]

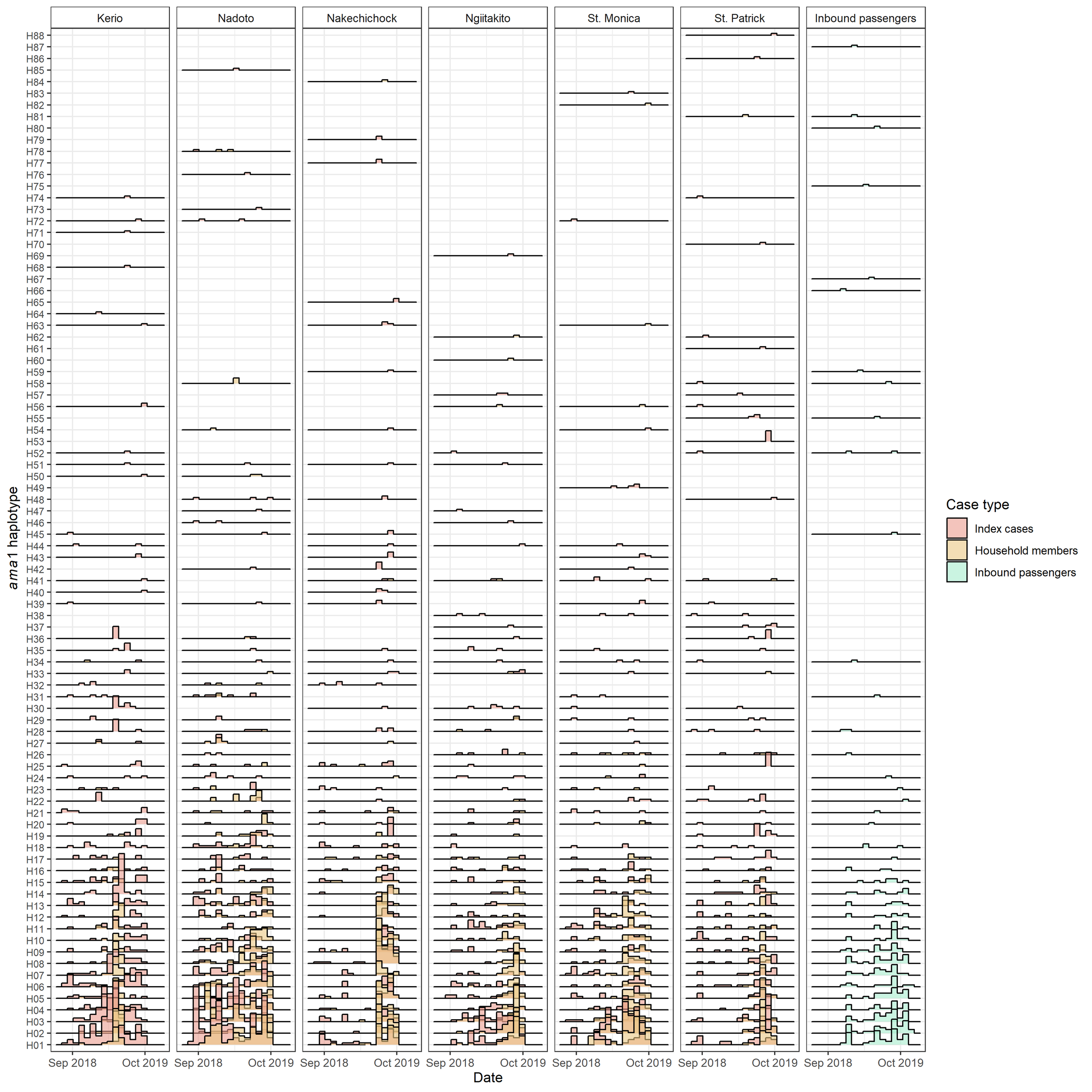

Supplement: S7 Fig — (TIF) [file pgph.0000807.s007.tif]

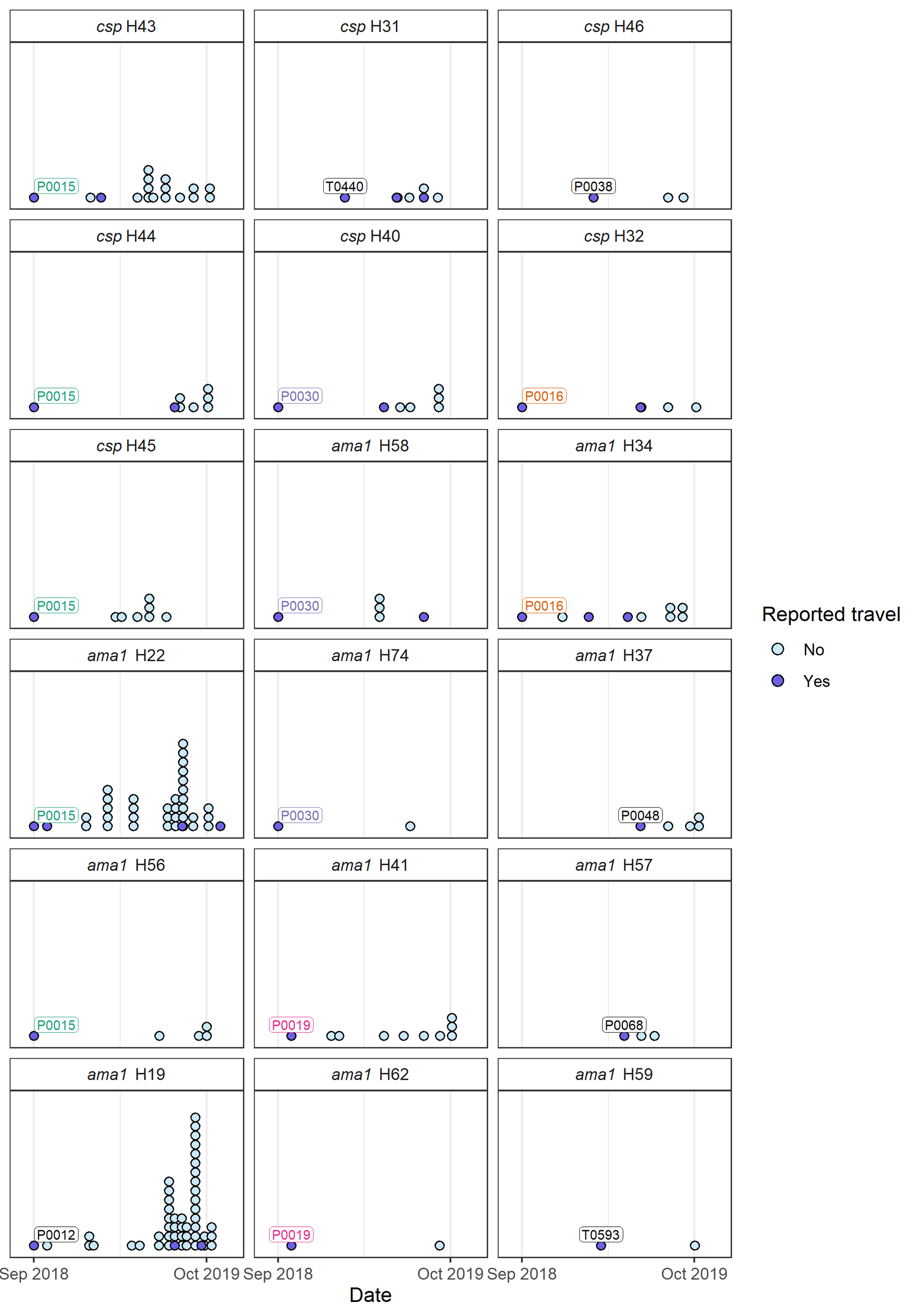

Supplement: S8 Fig — Seven csp haplotypes and 11 ama1 haplotypes were potentially imported into the study area by 10 individuals. All potential importations occurred either through inbound passengers or index cases reporting to the St. Patrick health facility. (TIF) [file pgph.0000807.s008.tif]

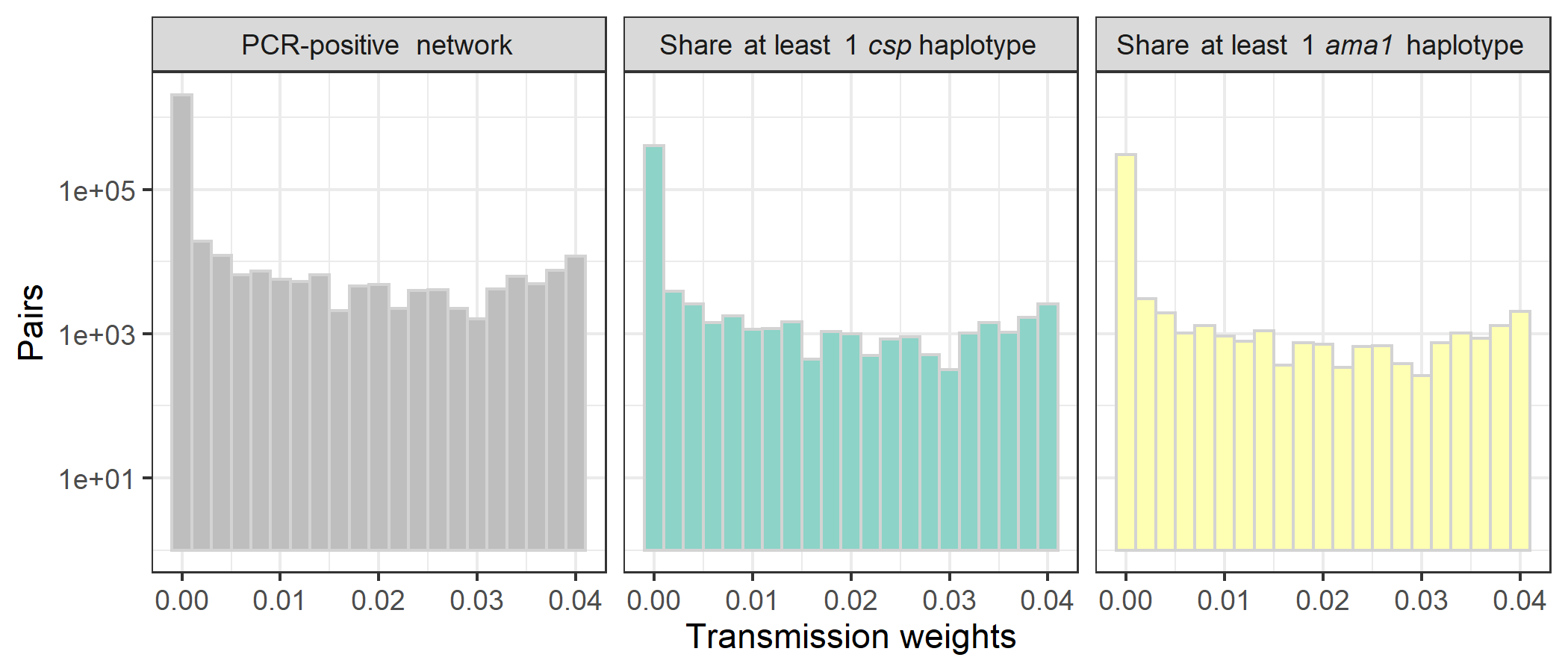

Supplement: S9 Fig — (TIF) [file pgph.0000807.s009.tif]

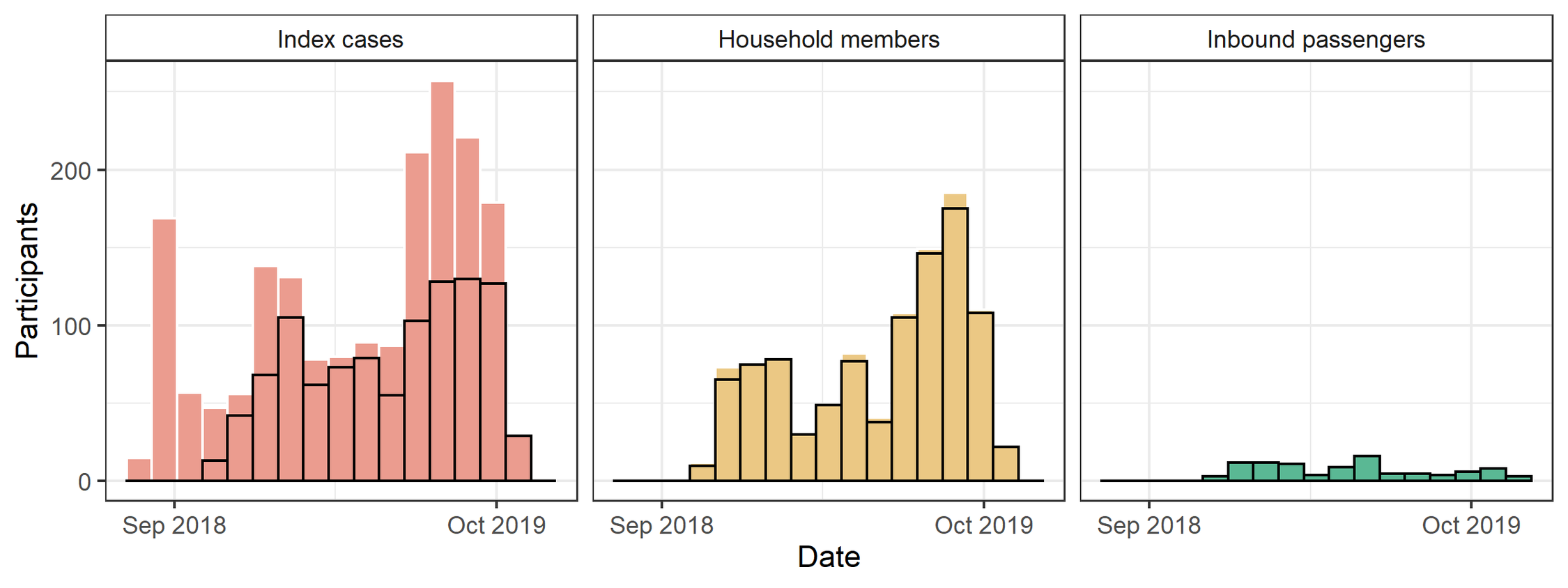

Supplement: S10 Fig — Because household members were assigned maximum transmission weights in the transmission network analysis, only those community participants for whom we had qPCR data for all household members were included in the analysis. Those included in the transmission analysis (black outline) are shown as a subset of all samples collected. (TIF) [file pgph.0000807.s010.tif]

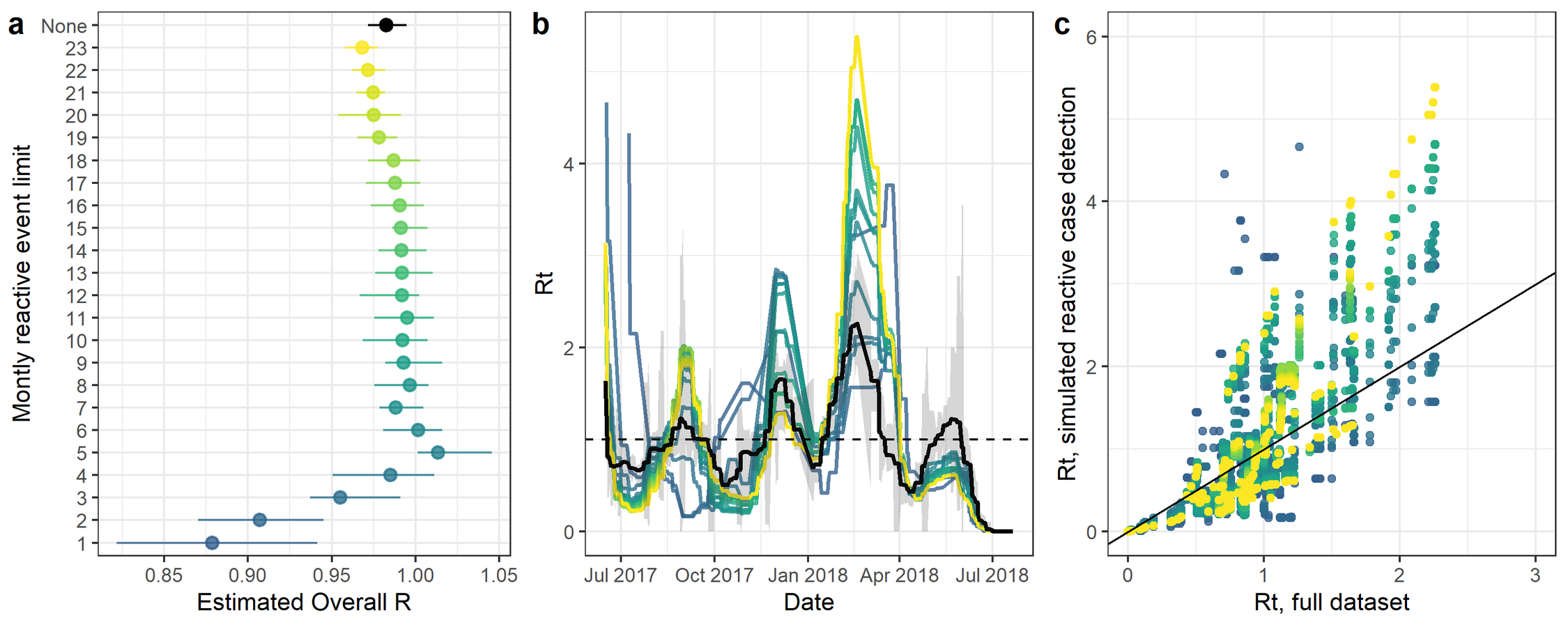

Supplement: S11 Fig — To understand how the reactive case detection (RCD) format of this study may have impacted estimated R values, we simulated RCD in a previously published household-based longitudinal cohort in Webuye, Kenya [31] with varying limits on the number of households investigated per month. RCD was simulated by identifying RDT-positive symptomatic malaria infections (simulated index cases) and sampling household member samples from the next monthly visit closest in time to the index case (simulated reactive case detection). Although (a) there were differences in the overall estimated R between the full longitudinal cohort and the simulated, RCD datasets (Welch ANOVA p < 0.0001), no systematic differences were observed when RCD events per month were capped as they were in the present study. Additionally, there was good correlation between (b and c) Rt estimates for the full dataset and for simulated RCD (Spearman rho 0.794, p < 0.0001). Black line in (c) is line of identity. (TIF) [file pgph.0000807.s011.tif]
