# Supplementary material for: Plasmodium falciparum importation does not sustain malaria transmission in a semi-arid region of Kenya
Source: PLOS Glob Public Health. 2022 Aug 10;2(8):e0000807. doi: 10.1371/journal.pgph.0000807 (PMC10021402; doi:10.1371/journal.pgph.0000807)
Supplement: S1 Table — (DOCX) [file pgph.0000807.s012.docx]

|  | Index cases  N = 1,891 | Household members  N = 3,314 | Inbound passengers  N = 1,891 | Overall  N = 7,096 |
| --- | --- | --- | --- | --- |
| Age (years) | | | | |
| ≤ 15 | 1,123 (60%) | 1,598 (49%) | 143 (7.6%) | 2,864 (41%) |
| 16 - 40 | 631 (34%) | 1,367 (42%) | 1,295 (69%) | 3,293 (47%) |
| > 40 | 124 (6.6%) | 326 (9.9%) | 441 (23%) | 891 (13%) |
| Gender |  |  |  |  |
| Male | 853 (46%) | 1,429 (43%) | 1,228 (65%) | 3,510 (50%) |
| Associated Health Facility | | | | |
| Rural | 1,147 (61%) | 2,070 (62%) |  | 3,217 (62%) |
| Kerio | 395 (21%) | 628 (19%) |  | 1,023 (20%) |
| Nadoto | 521 (28%) | 969 (29%) |  | 1,490 (29%) |
| Nakechichok | 231 (12%) | 473 (14%) |  | 704 (14%) |
| Urban | 744 (39%) | 1,244 (38%) |  | 1,988 (38%) |
| Ngiitakito | 272 (14%) | 465 (14%) |  | 737 (14%) |
| St. Monica | 265 (14%) | 467 (14%) |  | 732 (14%) |
| St. Patrick | 207 (11%) | 312 (9.4%) |  | 519 (10.0%) |
| *P. falciparum* RDT Positive | 1,891 (100%) | 394 (12%) | 44 (2.3%) | 2,329 (33%) |
| *P. falciparum* PCR Positive | 1,845 (98%) | 1,018 (31%) | 127 (6.7%) | 2,990 (42%) |
| Reporting any symptoms | 1,765 (99%) | 520 (16%) | 532 (19%) | 4,024 (58%) |
| Travel reported | 95 (5.0%) | 62 (1.9%) | 1,891 (100%) | 2,048 (29%) |
| n (%) |  |  |  |  |
