# Supplementary material for: Plasmodium falciparum importation does not sustain malaria transmission in a semi-arid region of Kenya
Source: PLOS Glob Public Health. 2022 Aug 10;2(8):e0000807. doi: 10.1371/journal.pgph.0000807 (PMC10021402; doi:10.1371/journal.pgph.0000807)
Supplement: S2 Table — (DOCX) [file pgph.0000807.s013.docx]

| Study ID | Case type | Gender | Age  group | Trips reported (n) | *Pf* PR_2-10_  Mean (SD)^a^ | MOI | | Haplotypes imported | | | |
| --- | --- | --- | --- | --- | --- | --- | --- | --- | --- | --- | --- |
|  |  |  |  |  |  | *ama1* | *csp* | *ama1* | *csp* | Total |  |
| P0015 | Index - St. Patrick | Male | 16-40 | 1 | 0.083 | 3 | 7 | 2 | 3 | 5 |  |
| P0030 | Index - St. Patrick | Male | 16-40 | 1 | 0.083 | 5 | 4 | 2 | 1 | 3 |  |
| P0016 | Index - St. Patrick | Female | < 5 | 1 | 0.513 | 1 | 1 | 1 | 1 | 2 |  |
| P0019 | Index - St. Patrick | Female | 16-40 | 2 | 0.083 (0.000) | 4 | 0 | 2 | 0 | 2 |  |
| P0012 | Index - St. Patrick | Male | 16-40 | 1 | 0.083 | 1 | 0 | 1 | 0 | 1 |  |
| P0048 | Index - St. Patrick | Female | 16-40 | 2 | 0.337 (0.250) | 2 | 0 | 1 | 0 | 1 |  |
| P0068 | Index - St. Patrick | Female | 16-40 | 1 | 0.404 | 5 | 0 | 1 | 0 | 1 |  |
| P0038 | Index - St. Patrick | Female | 16-40 | 1 | 0.179 | 0 | 1 | 0 | 1 | 1 |  |
| T0440 | Inbound passenger - visitor | Female | 16-40 | 2 | 0.202 (0.286) | 0 | 2 | 0 | 1 | 1 |  |
| T0593 | Inbound passenger - visitor | Male | 16-40 | 2 | 0.097 (0.032) | 1 | 0 | 1 | 0 | 1 |  |
| ^a^ *Pf* PR_2-10_ as for 2018-2019 from Malaria Atlas Project [26]. For inbound passengers visiting Turkana, the *Pf* PR_2-10_ reported is the mean of recent trips and residence. | | | | | | | | | | |  |
