# Supplementary material for: Plasmodium falciparum importation does not sustain malaria transmission in a semi-arid region of Kenya
Source: PLOS Glob Public Health. 2022 Aug 10;2(8):e0000807. doi: 10.1371/journal.pgph.0000807 (PMC10021402; doi:10.1371/journal.pgph.0000807)
Supplement: S3 Table — (DOCX) [file pgph.0000807.s014.docx]

| Marker | Haplotype | Not reporting travel (N) | Reporting travel (N) |
| --- | --- | --- | --- |
| *ama1* | H01 | 682 | 60 |
|  | H02 | 370 | 49 |
|  | H03 | 367 | 51 |
|  | H04 | 290 | 27 |
|  | H05 | 215 | 32 |
|  | H06 | 211 | 25 |
|  | H07 | 220 | 27 |
|  | H08 | 234 | 36 |
|  | H09 | 159 | 22 |
|  | H10 | 140 | 21 |
|  | H11 | 126 | 12 |
|  | H12 | 143 | 13 |
|  | H13 | 127 | 14 |
|  | H14 | 105 | 14 |
|  | H15 | 93 | 13 |
|  | H16 | 77 | 6 |
|  | H17 | 51 | 8 |
|  | H18 | 53 | 5 |
|  | H19 | 47 | 3 |
|  | H20 | 33 | 2 |
|  | H21 | 25 | 5 |
|  | H22 | 33 | 4 |
|  | H23 | 22 | 1 |
|  | H24 | 17 | 1 |
|  | H26 | 21 | 1 |
|  | H28 | 21 | 3 |
|  | H30 | 18 | 2 |
|  | H31 | 14 | 1 |
|  | H35 | 11 | 1 |
| *csp* | H01 | 829 | 72 |
|  | H02 | 599 | 57 |
|  | H03 | 346 | 45 |
|  | H04 | 288 | 31 |
|  | H05 | 337 | 29 |
|  | H06 | 183 | 14 |
|  | H07 | 152 | 24 |
|  | H08 | 138 | 13 |
|  | H09 | 232 | 30 |
|  | H10 | 102 | 8 |
|  | H11 | 101 | 9 |
|  | H12 | 79 | 9 |
|  | H13 | 65 | 5 |
|  | H14 | 92 | 5 |
|  | H15 | 64 | 5 |
|  | H16 | 66 | 4 |
|  | H18 | 37 | 1 |
|  | H19 | 32 | 2 |
|  | H20 | 38 | 5 |
|  | H21 | 33 | 2 |
|  | H22 | 20 | 3 |
|  | H23 | 38 | 8 |
|  | H24 | 26 | 4 |
|  | H25 | 19 | 1 |
|  | H26 | 28 | 4 |
|  | H27 | 17 | 2 |
|  | H28 | 14 | 1 |
|  | H30 | 11 | 1 |
|  | H34 | 13 | 1 |
|  | H43 | 15 | 2 |
|  | H48 | 13 | 1 |
