# Supplementary material for: Plasmodium falciparum importation does not sustain malaria transmission in a semi-arid region of Kenya
Source: PLOS Glob Public Health. 2022 Aug 10;2(8):e0000807. doi: 10.1371/journal.pgph.0000807 (PMC10021402; doi:10.1371/journal.pgph.0000807)
Supplement: Attachment — Submitted filename: Response to reviewers.pdf [file pgph.0000807.s016.pdf]

Duke | GLOBAL HEALTH INSTITUTE

Louisa Alexandra Messenger, MSc, PhD
Academic Editor, PLOS Global Public Health
London School of Hygiene & Tropical Medicine
London, United Kingdom

June 28, 2022

Dr. Messenger:

This letter accompanies the re-submission of our manuscript originally entitled “Importation of *Plasmodium falciparum* and sustained malaria transmission in a semi-arid region of Kenya” and now revised to “*Plasmodium falciparum* importation does not sustain malaria transmission in a semi-arid region of Kenya.” We would like to thank the reviewers for their suggested comments that have resulted in improvements to our original manuscript.

As with the prior submission, we certify that this manuscript is original and that it has not and will not be offered elsewhere for possible publication, as long as it is under consideration at *PLOS Global Public Health*. My co-authors Diana Menya, Amy Wesolowski, Daniel Esimit, Gilchrist Lokoel, Joseph Kipkoech, Elizabeth Freedman, Kelsey M. Sumner, Lucy Abel, George Ambani, Hannah R. Meredith, Steve M. Taylor, Andrew A. Obala, and Wendy P. O'Meara approved submission of this version of the manuscript.

Below, we have provided point-by-point responses to reviewer comments. For each comment, the reviewer comments are italicized and our responses are indented and in normal text, with specific changes underlined.

Reviewer 1

1. *The manuscript is well written and presented. It brings out an important aspect that needs to be addressed in many areas that are approaching elimination. However, there are minor comments / clarifications that need to be addressed. My comments are made in the submitted MS (attached).*

    RESPONSE: We have updated the manuscript to clarify the text based on your helpful suggestions. We have additionally answered the questions you posed in the points below.

2. *The title is slightly misleading and conveys a message that importation is the result of sustained transmission (post-importation) and hence may be revised to be a more result-oriented title.*

    RESPONSE: We agree and have therefore changed the title to “*Plasmodium falciparum* importation does not sustain malaria transmission in a semi-arid region of Kenya”

3. *Why wasn’t P. vivax studied? If studied, were there any mixed infections?*

RESPONSE: The scope of this study was limited to *P. falciparum* as indicated in the title and throughout the manuscript. To clarify the text and provide justification for why we only studied *P. falciparum*, we have added the following text to the manuscript:

Introduction lines 70-73: Consistent with these observations, we recently reported that nearly one-third of the household members of acute malaria patients in Central Turkana were also infected with *Plasmodium falciparum*, the major malaria parasite species in Kenya, suggesting that malaria is endemic in these communities.

Additionally, we have clarified in the methods that individuals were only screened for *P. falciparum* by RDT and PCR (lines 396, 403-403).

4. *Recommend to use a map to understand the geography and travel.*

RESPONSE: We have added the following map to the supplemental materials to help readers understand the geography and travel. (Figure S1)

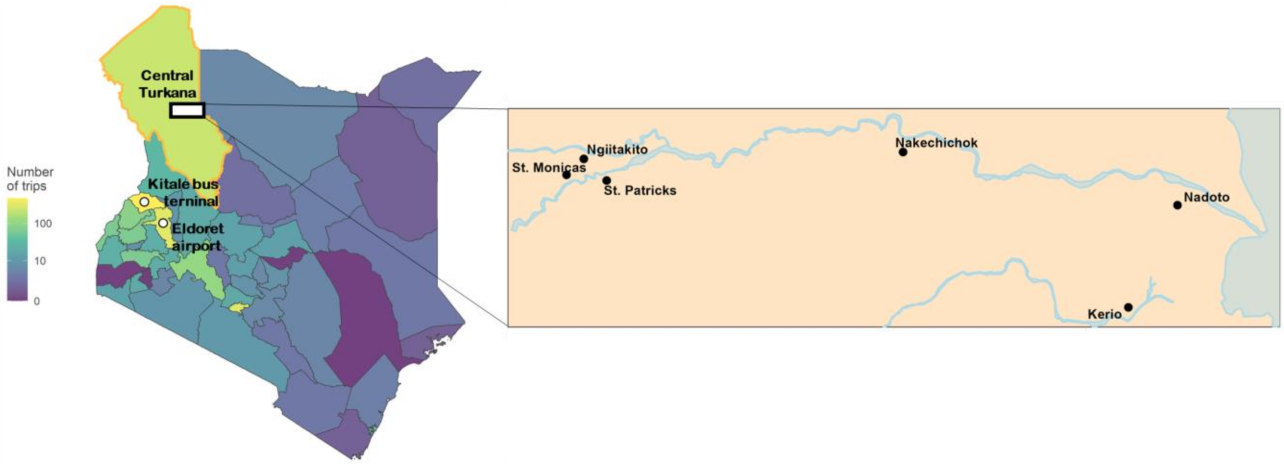

Figure S1. Map of Kenya with county borders. Turkana County is outlined in orange. County fill color denotes the number of trips to or from the designated county. The Kitale bus terminal and Eldoret airport are marked with white points. The study area in Central Turkana is highlighted with an inset map of health facilities to which index cases reported. Shapefile for the map of Kenya and county borders obtained from the Humanitarian Data Exchange (HDX), an open platform for sharing data across crises and organizations: https://data.humdata.org/dataset/cod-ab-ken . Inset map was created in QGIS (v 3.6.2-Noosa).

5. *What was the other (air) departure point and why was it skipped? Would have been better to screen the inbound travelers at Lodwar rather than at various departure points.*

RESPONSE: The omission of the other air departure point (Nairobi) was due to logistical study constraints. Although this is a limitation of our study, we feel that capturing half of the air departure points and the primary road travel to Lodwar presents a representative sample of inbound passengers to Central Turkana. We have added this to the discussion of limitations.

Lines 345-346:" Third, owing to logistical constraints, inbound passengers from only one of two air departure points to Lodwar Town were screened for *P. falciparum* malaria."

6. *Regarding AL treatment, was the intake supervised or they just "received" the drugs?*

    RESPONSE: All participants were provided with AL treatment at the point of RDT positivity. Intake was not supervised. We have clarified this in the methods section.

    Line 405-406: All RDT-positive participants received were provided with Artemether-Lumefantrine treatment (unsupervised), consistent with local guidelines for treatment.

Reviewer 2

7. *The data as presented is sufficient in showing parasite diversity in Turkana but there is no evidence to associate transmission with recent travel from neighboring areas of high transmission.*

    RESPONSE: As you correctly point out, the analyses presented here describe parasite diversity in Central Turkana and demonstrate that parasite importation does not sustain malaria transmission, but rather that *P. falciparum* is endemic in the region. These are the major findings of the paper and are important because historically Turkana has been considered inhospitable to malaria transmission (lines 66-68), and we have shown that there is significant, ongoing transmission in the region that is not fueled by travel. We have changed the title of the manuscript to better reflect this point.

8. *Figure 2 (b), shows low positivity rates in inbound passengers and thus cannot warranty imply malaria importation into Turkana County. There is possibility of inbound traveler getting malaria infection while in Turkana.*

    RESPONSE: You are correct - we observed low PCR positivity rates in inbound passengers (6.7%), particularly compared to household community members (30.7%). These finding support the major conclusion that inbound passengers are not sustaining malaria transmission in Central Turkana, as indicated by the title, abstract, and discussion in the manuscript.

9. *The mathematical model used herein to represent importation and transmission rates fails to consider pertinent variables like the traveler's malaria infection history, traveler's origin, whether the traveler got infection while in Turkana.*

    RESPONSE: The modified Wallinga Teunis model used in this study determined the relative likelihood that infected individuals are part of the same transmission chain and estimates the number of subsequent infections arising from a given infection (R) based on the time, distance, and haplotype matching between infection pairs. These distances included inbound passenger trip destinations. We accounted for participants' travel histories by running the model with and without individuals who reported travel in the 2 months prior to the study, and we found no significant differences in the estimated reproductive number between the two models. This is described in lines 237-243 of the manuscript:

“To quantify this, we then compared estimates of the reproductive number (R) in networks with and without people reporting travel in order to estimate the potential contribution of imported parasites to local transmission networks. We used a modified Wallinga Teunis model that incorporated time and distance into transmission weights for all possible infection pairs (**Figure S9**) and incorporated genotype information with increasing stringency under the assumption that infections which include the same haplotypes were more likely to be part of the same transmission network.”

10. *Turkana is a marginalized area where the inhabitants practice nomadic lifestyle, the author fails to establish how their lifestyle can also contribute in the importation of malaria parasites from neighboring counties.*

RESPONSE: <u>Lines 115 - 119</u> describe the trips reported by community members enrolled in our study in Central Turkana: “The 157 community-enrolled travelers reported 167 trips, 106 (67.5%) of which were within Turkana County. The majority of trips taken by community members outside of Turkana County were to counties with higher parasite prevalence based on Malaria Atlas Project (MAP) estimates (*Pf* $PR_{2\text{-}10}$ 2018-2019 [26]) (26/38 (68%) index case trips, 16/23 (70%) household member trips) (**Figure S2**).”

11. *The factor of climate change, vector abundance, and congruence of malaria parasites with other zoonotic protozoa needs to be considered in the arguments.*

RESPONSE: These are important factors for disease transmission, though they fall outside the scope of this study. However, we have included some discussion of the ecological factors of the study area in the discussion section, copied below.

<u>Lines 354-367</u>: “Like other non-traditional settings adjacent to malaria hyperendemic regions, Turkana County has historically been outside the malaria risk map in Kenya and therefore overlooked for country-wide interventions and control measures [15]. Such preventive measures typically include distributions of insecticide-treated bed nets and vector control with indoor residual spraying or other approaches. These are rare in Turkana County [18], though their effectiveness may be attenuated by the unique ecological and epidemiologic features of the region, which include little surface water with only evanescent vector breeding sites, a (semi-)nomadic population, and limited opportunities for bed net use. Among the potential measures for transmission reduction would be prevention of importation, but our finding that malaria is not only prevalent but also sustained by local transmission suggests that interrupting importation is not likely to be an effective malaria control strategy on its own. Spatio-temporal and household-based genetic relatedness of infections, combined with understanding of the unique ecology of the region, can be used to test which interventions may be most effective at reducing transmission in Turkana County.”

<u>Reviewer 3</u>

12. *Tableau: the “N” for sample size should be “n” as they sampled from the population. “N” would mean the entire population.*

RESPONSE: Thank you. We have changed the table accordingly.

13. *Figure 3. There are some haplotypes that are unique to specific enrollment sites. For instance, the haplotype H55, H63, and H60 are only seen in St. Patrick. What is the particularity of this site? This should be discussed in the manuscript.*

    RESPONSE: There are a number of *csp* (18/69, 26%) and *ama1* (24/83, 29%) haplotypes which were only detected at a single health facility. These haplotypes tended to have lower frequencies in the population overall (Fig S6 and S7). Our sampling strategy, though robust, did not capture all infections in Central Turkana, so it is possible that infections with these haplotypes occurred in the catchment area of more than one facility, but we did not detect them due to their low population frequencies. It is also possible that *de novo* mutations would lead to haplotypes that are unique to individual study sites.

14. *Page 8, line 212: figure 4c is listed before figure 4a and 4b. This should be corrected. The figure 4c should be figure 4a.*

    RESPONSE: Thank you for noticing this detail. We have re-ordered Figure 4 to reflect the order in which the panels are discussed in the text.

15. *Throughout the manuscript, Central Turkana and Turkana are used interchangeably. Are they the same? If yes, please use only one as it is confused.*

    RESPONSE: Turkana refers to Turkana County, and Central Turkana refers to the study area. We have revised the text to make this distinction more clear. We have also added a map (Figure S1) to the supplemental information designating the study area of Central Turkana.

16. *Even though, the enrollment sites are well-described in the method section, a map in the supplemental material indicating these sites would be more informative for readers.*

    RESPONSE: Thank you for this suggestion. As discussed in #4 and #15 above, we have added a map to the supplemental information (Fig S1).

17. *Make the ama1, csp sequence data available through a public repository or as a fasta file.*

    RESPONSE: Sequences from this study have been deposited as a Bioproject in the NIH Short Read Archive. (BioProject PRJNA850951).

Thank you for your consideration of our manuscript.

Sincerely,

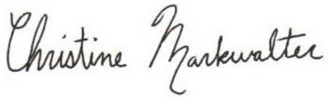

Christine Markwalter, Ph.D.

Duke Global Health Institute | Box 90519, Duke University, Durham NC 27708
919-681-7760 | www.globalhealth.duke.edu
